# Thermostability of Hybrid Thermoelectric Materials Consisting of Poly(Ni-ethenetetrathiolate), Polyimide and Carbon Nanotubes

**DOI:** 10.3390/ma10070824

**Published:** 2017-07-18

**Authors:** Keisuke Oshima, Shifumi Sadakata, Hitoshi Asano, Yukihide Shiraishi, Naoki Toshima

**Affiliations:** 1 Graduate School of Engineering, Tokyo University of Science Yamaguchi, Daigakudori, SanyoOnoda, Yamaguchi 756-0884, Japan; f115701@ed.tusy.ac.jp; 2 Department of Applied Chemistry, Faculty of Engineering, Tokyo University of Science Yamaguchi, Daigakudori, SanyoOnoda, Yamaguchi 756-0884, Japan; jf312020@ed.tus.ac.jp (S.S.); asano@rs.tusy.ac.jp (H.A.); 3 Department of Applied Chemistry, Tokyo University of Science, Yamaguchi 756-0884, Japan; toshima@rs.tus.ac.jp

**Keywords:** organic/inorganic hybrid thermoelectric materials, polyimide, poly(nickel 1,1,2,2-ethenetetrathiolate), carbon nanotube

## Abstract

Three-component organic/inorganic hybrid films were fabricated by drop-casting the mixed dispersion of nanodispersed-poly(nickel 1,1,2,2-ethenetetrathiolate) (nano-PETT), polyimide (PI) and super growth carbon nanotubes (SG-CNTs) in *N*-methylpyrrolidone (NMP) at the designed ratio on a substrate. The dried nano-PETT/PI/SG-CNT hybrid films were prepared by the stepwise cleaning of NMP and methanol, and were dried once more. The thermoelectric properties of Seebeck coefficient *S* and electrical conductivity *σ* were measured by a thin-film thermoelectric measurement system ADVANCE RIKO ZEM-3M8 at 330–380 K. The electrical conductivity of nano-PETT/PI/SG-CNT hybrid films increased by 1.9 times for solvent treatment by clearing insulated of polymer. In addition, the density of nano-PETT/PI/SG-CNT hybrid films decreased 1.31 to 0.85 g·cm^−3^ with a decrease in thermal conductivity from 0.18 to 0.12 W·m^−1^·K^−1^. To evaluate the thermostability of nano-PETT/PI/SG-CNT hybrid films, the samples were kept at high temperature and the temporal change of thermoelectric properties was measured. The nano-PETT/PI/SG-CNT hybrid films were rather stable at 353 K and kept their power factor even after 4 weeks.

## 1. Introduction

Energy is a key issue for human beings. The increasing world population, food shortages, and economic differences are also big issues, but these problems could be solved if we had enough sustainable energy. Presently, developed countries as well as developing countries obtain most of their energy from fossil fuels like coal, petroleum and natural gas. When we use these energy sources, more than half of the energy is lost without utilizing them in the planned form, which results in waste heat. For example, we use most of our energy in the form of electricity. In 2014, the percentage of electricity in total energy was 25.3% in Japan. However, the conversion efficiency to get electricity from fossil fuels was 42.2% in Japan in 2014, meaning that 57.8% of the energy from fossil fuels was lost as heat energy without its use. If we could acquire some electricity from these lost heat energies, we could reduce the consumption of fossil fuels and reduce the increasing worldwide temperature. For this reason, thermoelectric technology has become interesting in recent years.

The utilization of unused heat energy like natural heat and waste heat below 423 K has received much attention recently. About two thirds of the waste heat is at a low grade, i.e., below 423 K [1]. The efficiency of a thermoelectric material depends on a dimensionless figure of merit, *ZT* [2], calculated according to Equation (1):

*ZT* = *S*^2^*σT*/*κ* (1)

where *S* is the Seebeck coefficient (V·K^−1^), *σ* is the electrical conductivity (S·m^−1^), *T* is the absolute temperature (K), and *κ* is the thermal conductivity (W·m^−1^·K^−1^). If materials have similar thermal conductivities, the parameter power factor, *PF* [3] (=*S*^2^*σ*) is used to characterize thermoelectric performances. Thus, good thermoelectric materials possess large power factors and low thermal conductivities.

Inorganic thermoelectric materials, such as Mg_2_Si, and Bi_2_Te_3_, etc., have been the focus of research [4,5], while research related to organic thermoelectric materials [6,7,8] has received less attention, mainly because of their low figures of merit [9,10]. However, inorganic materials have several disadvantages, such as rarity, high cost in production, poor processability, and environmental problems due to their toxicity. On the other hand, organic thermoelectric materials, compared with inorganic thermoelectric materials, have many advantages, such as plenty of raw resources, easy processability into a versatile form, easy application of printing technology to fabricate devices with large areas, low cost in raw materials and device manufacturing, environmental friendliness, and so on. The most exciting results on organic thermoelectric materials involve thin films of polyphenylenevinylene (PPV) derivatives after stretching [11], poly(3,4-ethylene dioxythiophene) *p*-toluenesulfonate (PEDOT-Tos) at controlled oxidation level [12], and poly(3,4-ethylenedioxythiophene) poly(styrenesulfonate) (PEDOT-PSS) [13,14,15] after ethylene glycol treatment. Although devices made from PEDOT-based films show high performance as thermoelectric materials, their instability in an ambient environment is a serious disadvantage that limits their practical use. It is difficult to achieve higher thermoelectric performances using only organic materials such as conducting polymers [16,17,18].

Recently, hybrid materials using organic and inorganic materials have been studied for next-generation thermoelectric materials. The properties of novel hybrid types based on organic thermoelectric materials are different from those of inorganic thermoelectric materials, and it is therefore possible to enhance electrical conductivity and to reduce thermal conductivity. For example, hybrid organic materials show good thermoelectric performance by incorporating organic materials with carbon nanotubes (CNTs) [19,20,21,22], nanocrystals [23,24,25,26], or nanoparticles [27,28,29] as inorganic materials. CNTs were thought not to be very good thermoelectric materials because of their high thermal conductivity [30]. However, CNTs with high Seebeck coefficients were reported to have a good thermoelectric performance [31]. In addition, CNTs with organic ligands, such as tetraphenylphosphine, were recently demonstrated to have a relatively high negative Seebeck coefficient and low thermal conductivity, resulting in *ZT* = 0.07 [32]. Preliminarily, we have successfully developed new thermoelectric materials with high thermoelectric property by using nanodispersed-poly(Ni 1,1,2,2-ethenetetrathiolate) (nano-PETT), poly(vinylchloride) (PVC), and CNTs [33]. The nanoparticles of polymer complexes, nano-PETT, were able to be well dispersed in PVC hybrids, and played an important role for the smooth contact and good charge transport between CNT bundles in the films. In these cases, however, PVC was also not stable at high temperature. Thus, development of thermostable hybrid thermoelectric materials is strongly required for fabrication of thermoelectric devices. In this study, organic/inorganic hybrid thermoelectric materials were prepared using a heat resistant polymer; polyimide (PI), nano-PETT, and SG-CNTs.

## 2. Materials and Experimental Processes

### 2.1. Materials

To synthesis nano-PETTs, we used 1,3,4,6-tetrathiapentalene-2,5-dione (TPD, Tokyo Chemical Industry Co., Ltd., Tokyo, Japan). Nickel (II) were selected as center metals (Wako Pure Chemical Ind., Ltd., Hiroshima, Japan). Polyimide (PI, TS-8, Solpit Industries Co., Ltd., Tokyo, Japan) for binder was used. SG-CNTs (Zeon Corp., Tokyo, Japan) were used to improve electrical conductivity and mechanical properties of polymer.

### 2.2. Preparation of Nano-PETT

When the powders of the solid PETT, prepared according to the literature [34], were sonicated in NMP containing the surfactant for an hour, the solid Ni-PETT could not be dispersed in it at all. Thus, we used the surfactant during the synthesis of nano-PETT as follows: dodecyltrimethyl-ammonium bromide (6.9 g, 22.2 mmoL) and sodium methoxide (1.2 g, 22.2 mmoL) were dissolved in methanol (190 mL) by stirring the mixtures with a magnetic stirrer at room temperature under air overnight to produce a pale yellow solution. To this solution, 1,3,4,6-tetrathiapentalene-2.5-dione (1.0 g, 4.8 mmoL) was added. The mixture was refluxed for 12 h at 363 K in an oil bath and kept in the oil bath overnight after the refluxing. The color of the solution gradually changed from yellow to black. Then, nickel (II) chloride (4.8 mmoL) and methanol (10 mL) were added to this mixture. The mixture was refluxed for another 12 h, and then gradually cooled down by keeping the flask in an oil bath overnight. The produced black precipitates were separated by suction filtration membrane filter (polytetrafluoro-ethylene with pore size 0.1 µm, Toyo Roshi Kaisha Ltd., Tokyo, Japan), washed with 2 L water, methanol and a small amount of diethyl ether, and then dried under vacuum at 313 K through a night. The obtained black powders of surfactant-containing nano-PETT were able to be dispersed in NMP to produce a brown solution. The majority of the nano-PETT was in the range of 10–50 nm, suggesting that the size of nano-PETT is nearly homogeneous [35]. The results reveal that nano-PETT examined here has an average diameter of 37.6 nm.

### 2.3. Fabrication of Three-Component Hybrid Films: Nano-PETT/PI/SG-CNT

The nano-PETT and SG-CNTs were added to an NMP solution containing PI, and dispersed in an ultrasonic homogenizer for 10 min. The dispersions of nano-PETT, PI, and SG-CNT were cast on a petri dish and/or polyimide substrate, and dried in air on a hot plate at 333 K for 12 h to obtain a three-component hybrid film, nano-PETT/PI/SG-CNT.

### 2.4. Characterization

The film thickness was measured with a linear gage (model LGK-010, resolution: 0.1 μm, Mitsutoyo Corp., Kawasaki, Japan). Seebeck coefficient and electrical conductivity of nano-PETT/PI/SG-CNT were measured with a thermoelectric evaluation system (ZEM-3M8, ADVANCE RIKO, Inc., Yokohama, Japan) at 330–380 K under vacuum with helium gas. The values of the power factor for each of the films were calculated by the equation *PF* = *S*^2^*σ*. The surface morphology of the film was observed with a field emission scanning electron microscope (FE-SEM, S-4800 Type2, Hitachi, Tokyo, Japan). Thermal conductivity, *κ*, was calculated by Equation (2)

*κ* = *αρC*_p_ (2)

where *α* is the thermal diffusivity, *ρ* is the density calculated by measuring the weight and volume of the films, and *C*_p_ is the specific heat capacity at constant pressure measured using a Netzsch DSC 204 F1 Phoenix (Yokohama, Japan). The thermal diffusivity *α* was measured with a Netzsch LFA 447 Nanoflash (Yokohama, Japan) in a through-plane direction of self-standing films at 290 K.

## 3. Results and Discussion

### 3.1. Preparation and Characterization of Nano-PETT/PI/SG-CNT Hybrid Films

**Figure 1:**
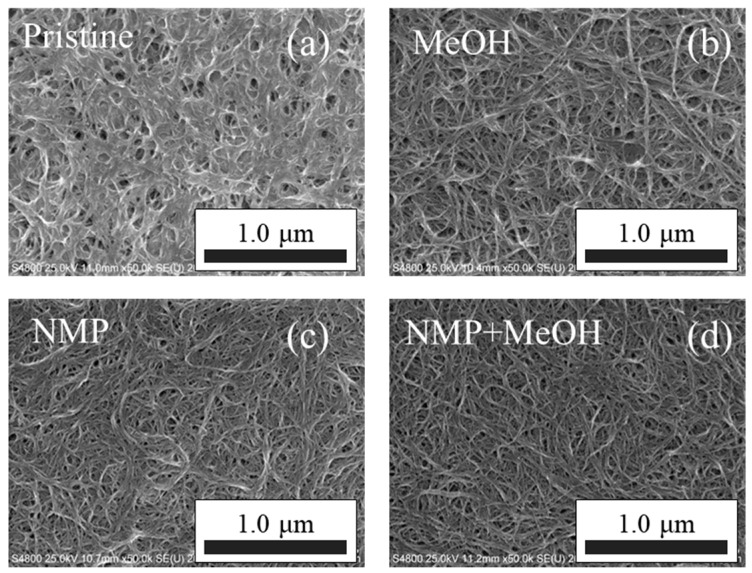
SEM images of the hybrid films of nano-PETT/PI/SG-CNT before cleaning (**a**), after methanol cleaning (**b**); after *N*-methylpyrrolidone (NMP) cleaning (**c**) and after the stepwise cleaning by NMP and methanol (**d**).

Maniwa et al. reported that purified semiconducting single-walled CNTs had a very high Seebeck coefficient of 170 μV·K^−1^ at 350 K, which would make them suitable for use in flexible thermoelectric materials [31]. On the other hand, the SG-CNTs have many defects, and a poor thermoelectric performance by themselves. In order to improve the performance of SG-CNTs, three-component nano-PETT/PI/SG-CNT hybrid films were prepared by a conventional drop-casting method from a mixed dispersion of nano-PETT, PI and SG-CNTs in NMP at the designed ratios on a petri dish. The SEM images of nano-PETT/PI/SG-CNT hybrid films before and after solvent treatment are shown in Figure 1. The SEM images of nano-PETT/PI/SG-CNT hybrid films before cleaning (Figure 1a) and after methanol cleaning (Figure 1b) reveal that they are composed of complicated large masses. We previously found that treatment of the organic/inorganic hybrid films with methanol could enhance the electrical conductivity [35]. In these nano-PETT/PI/SG-CNT hybrid films, however, the solubility of PI in methanol was poor. When NMP was used instead of methanol, the nano-PETT/PI/SG-CNT hybrid films exhibited good film. Furthermore, the SEM image of nano-PETT/PI/SG-CNT hybrid films after the stepwise cleaning by NMP and methanol (Figure 1d) showed few CNT bundles. The film’s thickness decreased from 52.7 ± 2.6 µm to 31.0 ± 1.6 µm.

We next measured the thermal conductivity of the nano-PETT/PI/SG-CNT hybrid films in the through-plane direction. Recently, anisotropy in thermal conductivity has become a hot topic in organic and hybrid thermoelectric materials [36]. Since the Seebeck coefficient and electrical conductivity were measured in the in-plane direction, the thermal conductivity needs to be measured in the same direction in order to obtain the correct thermoelectric figure-of-merit. However, it is difficult to obtain the correct thermal conductivity of the thin films in the in-plane direction. The data used to calculate the thermal conductivity of the nano-PETT/PI/SG-CNT hybrid films before and after solvent cleaning are summarized in Table 1. The density of nano-PETT/PI/SG-CNT hybrid films decreased 1.31 to 0.85 g·cm^−3^, with a decrease in thermal conductivity from 0.18 to 0.12 W·m^−1^·K^−1^ before and after the stepwise cleaning by NMP and methanol, respectively. It should be emphasized that the solvent treatment provides a low thermal conductivity, *κ*, which could be due to the low density, *ρ*. It is clearly demonstrated by the SEM photographs shown in Figure 1d that the films became porous after the stepwise cleaning by NMP and methanol, which could result in many voids and low density. Another reason for the low thermal conductivity of the nano-PETT/PI/SG-CNT hybrid films is the utilization of PI, the thermal conductivity of which is intrinsically low.

### 3.2. Thermoelectric Properties of Nano-PETT/PI/SG-CNT Hybrid Films

**Figure 2:**
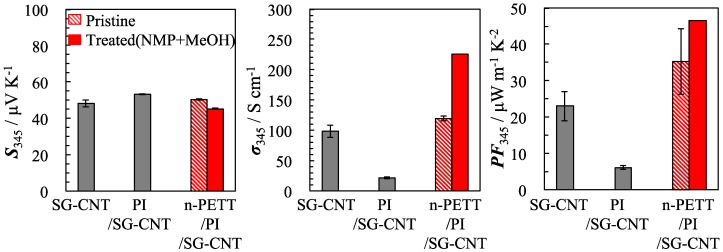
Seebeck coefficient (*S*), electrical conductivity (σ) and power factor (*PF*) of SG-CNT sheet, PI/SG-CNT and nano-PETT/PI/SG-CNT. Mass ratios are PI/SG-CNT = 12/8, and nano-PETT/PI/SG-CNT = 9/3/8. All films have a thickness of 10 µm.

The thermoelectric properties of the sheet of SG-CNT, PI/SG-CNT and nano-PETT/PI/SG-CNT used in this research are shown in Figure 2. The Seebeck coefficients of SG-CNT, PI/SG-CNT and nano-PETT/PI/SG-CNT hybrid films (pristine) were 48 μV·K^−1^, 53 μV·K^−1^, and 50 μV·K^−1^, respectively. The Seebeck coefficient did not depend on the type of sheet. The CNTs themselves had an electrical conductivity of ca. 98 S·cm^−1^. When they were dispersed in PI, however, the electrical conductivity of the hybrids of PI/SG-CNT was as low as 22 S·cm^−1^. In contrast, the electrical conductivity of nano-PETT/PI/SG-CNT hybrid films was higher than that of PI/SG-CNT films. In addition, we found that treatment of the nano-PETT/PI/SG-CNT hybrid films by stepwise cleaning by NMP and methanol could enhance the electrical conductivity. A similar enhancement in electrical conductivity by treatment with a solvent such as DMSO and EG has been reported in the case of PEDOT films, where the solvent treatment was considered to enhance the alignment of the conducting polymer chains [37] and/or to remove the insulating materials from the surface of the films [13]. The electrical conductivity of nano-PETT/PI/SG-CNT hybrid films increased 1.9 times for solvent treatment by clearing insulated of polymer. The electrical conductivity is known to be proportional to the carrier concentration and carrier mobility. The addition of the nano-PETT increased the electrical conductivity, while the Seebeck coefficient remained constant. This means that the nano-PETT does not work as an electron conductor, but covers the defects of the SG-CNTs, which results in an increased electron mobility [38], although the real mobility could not be measured for technical reasons. Hereby, the power factor of nano-PETT/PI/SG-CNT hybrid films is ca. 2.0 times higher than that of SG-CNT. We previously found that the treatment of organic/inorganic hybrid films could enhance the power factor [39]. When poly(methyl methacrylate) was employed as a binder, poly(methyl methacrylate) hybrid films were very fragile after methanol treatment. It is important to select an optimal polymer, because the polymer added as a binder affects film properties [40]. Thus, it is striking that PI is very effective for obtaining high thermoelectric properties for the hybrid films.

**Figure 3:**
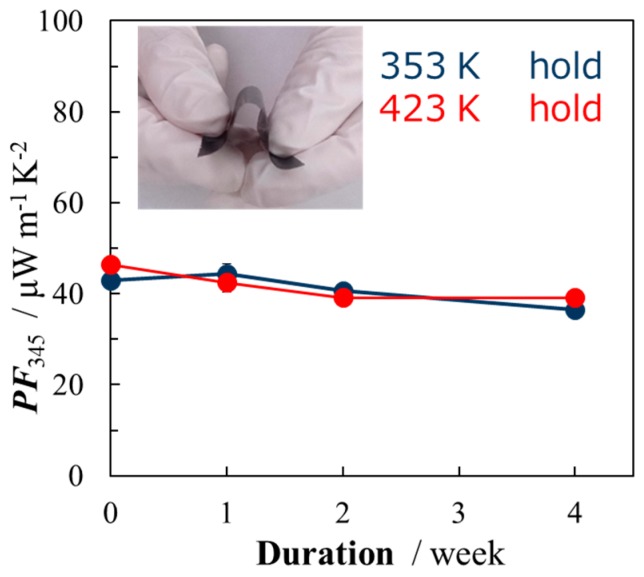
Durability of power factor (*PF*) at 353 K and 423 K of nano-PETT/PI/SG-CNT hybrid films (film thickness of 10 μm).

**Figure 4:**
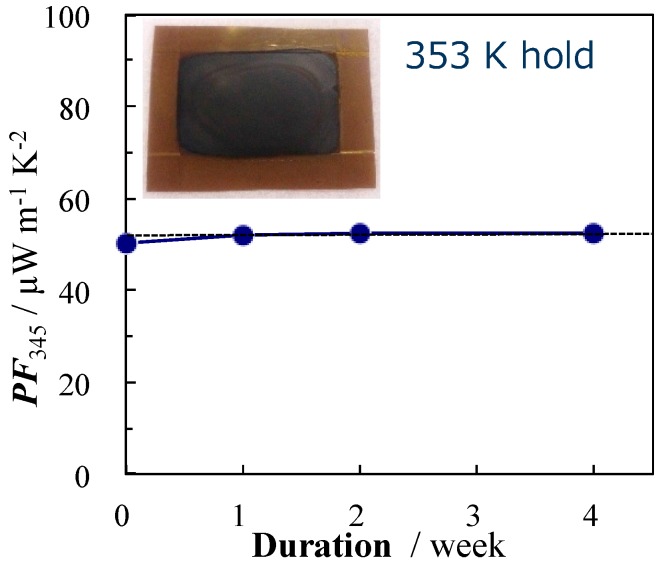
Durability of power factor (*PF*) at 353 K of nano-PETT/PI/SG-CNT hybrid films (film thickness of 3 μm).

In order to evaluate the thermal long-term stability of nano-PETT/PI/SG-CNT hybrid films (film thickness of 10 μm), samples were kept at 353 K and 423 K, and the temporal change of the thermoelectric properties in air was measured for 4 weeks. The relationship between duration and the power factor of nano-PETT/PI/SG-CNT hybrid films is shown in Figure 3. The power factor of nano-PETT/PI/SG-CNT hybrid films at 353 K decreased from 46 to 39 μW·m^−1^·K^−2^. The percentage of weight loss from 0 day to 4 weeks at 353 K was −15%. Moreover, the percentage of weight loss from 0 day to 4 weeks was −16% even at the higher temperature of 423 K. This is probably because PI may protect nano-PETT from the decomposition at the higher temperature. We next prepared the nano-PETT/PI/SG-CNT hybrid films with a film thickness of 3 μm by coating the mixed dispersion of nano-PETT, PI and SG-CNTs in NMP on a polyimide substrate, as shown in Figure 4 (inset photograph). Figure 4 depicts the relationship between duration and the power factor of these thin films. These thin nano-PETT/PI/SG-CNT hybrid films were rather stable at 353 K, and kept their power factor even after 4 weeks.

**Figure 5:**
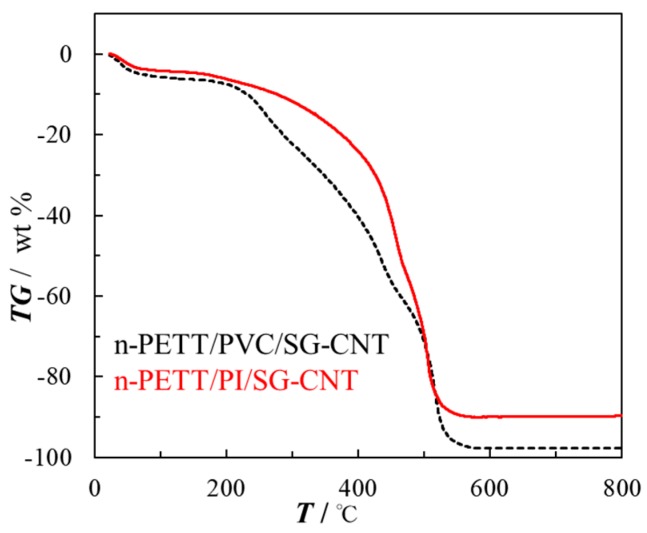
Comparison of thermogravimetric analyses of nano-PETT/PI/SG-CNT (red line) and nano-PETT/PVC/SG-CNT (black line) hybrid films. Mass ratio: nano-PETT/polymer/SG-CNT = 9/3/8.

In order to examine the thermostability of nano-PETT/PI/SG-CNT hybrid films, thermogravimetric analyses were performed under air. A thermal decomposition temperature of nano-PETT/PI/SG-CNT hybrid films (695 K) was higher than that of nano-PETT/PVC/SG-CNT hybrid films (476 K), as shown in Figure 5. The thermal decomposition temperatures of nano-PETT, PI, and SG-CNT are 413 K, 825 K, and 854 K, respectively. In spite of the low decomposition temperature of nano-PETT, thermal decomposition was suppressed in the nano-PETT/PI/SG-CNT hybrid films. Previously, we reported that poly(sodium acrylate)(PAA)-protected Ag nanoclusters are much more stable than poly(*N*-vinyl-2-pyrrolidone)(PVP)-protected Ag nanoclusters, especially at high temperature [41]. The increment of weight loss between PAA and PAA-Ag nanoclusters at 443 K (1.9%) was much smaller than that between PVP and PVP-Ag nanoclusters (3.8%). The protection polymer plays an important role not only in protecting nanomaterials, but also in controlling functions [42,43]. In nano-PETT/PI/SG-CNT hybrid films, PI is the significant material, not only for binder but also for long-term thermal stability. One of the biggest of the various advantages of hybrid thermoelectric materials is the low cost of fabrication for hybrid thermoelectric devices. It might be possible that these nano-PETT/PI/SG-CNT hybrid films allow the fabrication of devices by simple methods like printing.

## 4. Conclusions

In summary, we have developed a three-component organic/inorganic hybrid material of nano-PETT/PI/SG-CNT with high thermoelectric properties. The Seebeck coefficient and the electrical conductivity of nano-PETT/PI/SG-CNT were 45 μV·K^−1^ and 226 S·cm^−1^ at 345 K, respectively. The materials exhibit a high *PF* value of 46 μW·m^−1^·K^−2^ at 345 K. The nano-PETT/PI/ SG-CNT hybrid films are rather stable at 353 K, and retain a negative power factor even after 4 weeks. The stabilization of the nano-PETT/PI/SG-CNT hybrid films can be understood by the polymer effect of polyimide surrounding SG-CNTs. The developed nano-PETT/PI/SG-CNT hybrid films are expected to be a candidate as hybrid materials for use in future thermoelectric devices, which could have advantages in terms of flexibility and long lifetime. For energy harvesting purposes, the thermoelectric devices could be used to provide electricity in dark places, as the power sources of sensors instead of batteries. This novel concept of the role of functional polymers may open a new field in organic/inorganic hybrid electronics.

## Figures and Tables

**Table 1:** Thermal conductivity *κ* and related data of nano-PETT/PI/SG-CNT hybrid films at 290 K.

	Pristine	Treated		
		MeOH	NMP	NMP + MeOH
***ρ***/g·cm^−3^	1.31 ± 0.06	1.12 ± 0.15	1.12 ± 0.08	0.85 ± 0.04
***α***/mm^2^·s^−1^	0.13 ± 0.03	0.14 ± 0.00	0.13 ± 0.01	0.15 ± 0.00
***C*_p_**/J·g^−1^·K^−1^	0.86 ± 0.06	0.87 ± 0.01	0.80 ± 0.02	0.93 ± 0.04
***κ***/W·m^−1^·K^−1^	0.18 ± 0.01	0.14 ± 0.02	0.12 ± 0.01	0.12 ± 0.02
